# Supplementary material for: Solid-Phase Microextraction/Gas Chromatography–Time-of-Flight Mass Spectrometry Approach Combined with Network Pharmacology Analysis to Evaluate the Quality of Agarwood from Different Regions against Anxiety Disorder
Source: Molecules. 2024 Jan 17;29(2):468. doi: 10.3390/molecules29020468 (PMC10818993; doi:10.3390/molecules29020468)
Supplement: Supplementary file 1 [file molecules-29-00468-s001.zip › molecules-2601240-supplementary.pdf]

Supplementary Material

Figure S1. Representative total ion chromatograms (TIC) of agarwood samples from four geographical origins.

Figure S2. PCA (a) and PLS-DA (b) score plots of agarwood samples from different geographical origins based on their detected metabolites using SPME-GC/TOFMS (n = 10). Yellow circle, Malaysia group; Red diamond, Brunei group; Purple triangle, Irian group; Green inverted triangle, Nha Trang group.

Figure S3. Overlapping genes (79) between those (1,942) related to anxiety disorders and those (254) related to the differential sesquiterpenes in agarwood.

Figure S4. Histogram of selected compounds that are significantly different among agarwood groups. (A) Brunei group; (B) Nha Trang group; (C) Malaysia group; (D) Irian group. Data are presented as the mean ± SD (n=10). * $p < 0.05$, ** $p < 0.01$, *** $p < 0.001$.

Table S1. List of the detected volatile compounds in agarwood samples.

Table S2. Sixty-two sesquiterpenes with significant differences among agarwood groups (PLS-DA VIP > 1.0 and ANOVA $p < 0.05$).

Table S3. Agarwood samples analyzed in the present study.

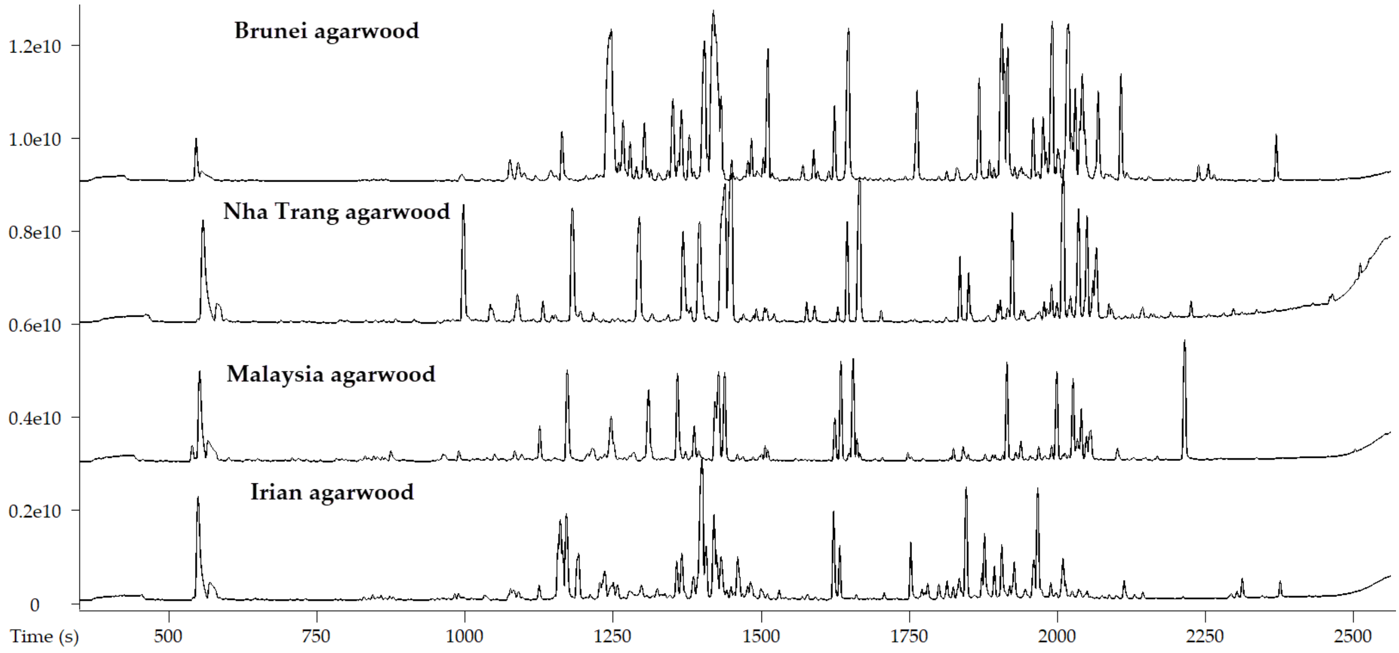

Figure S1. Representative total ion chromatograms (TIC) of agarwood samples from four geographical origins.

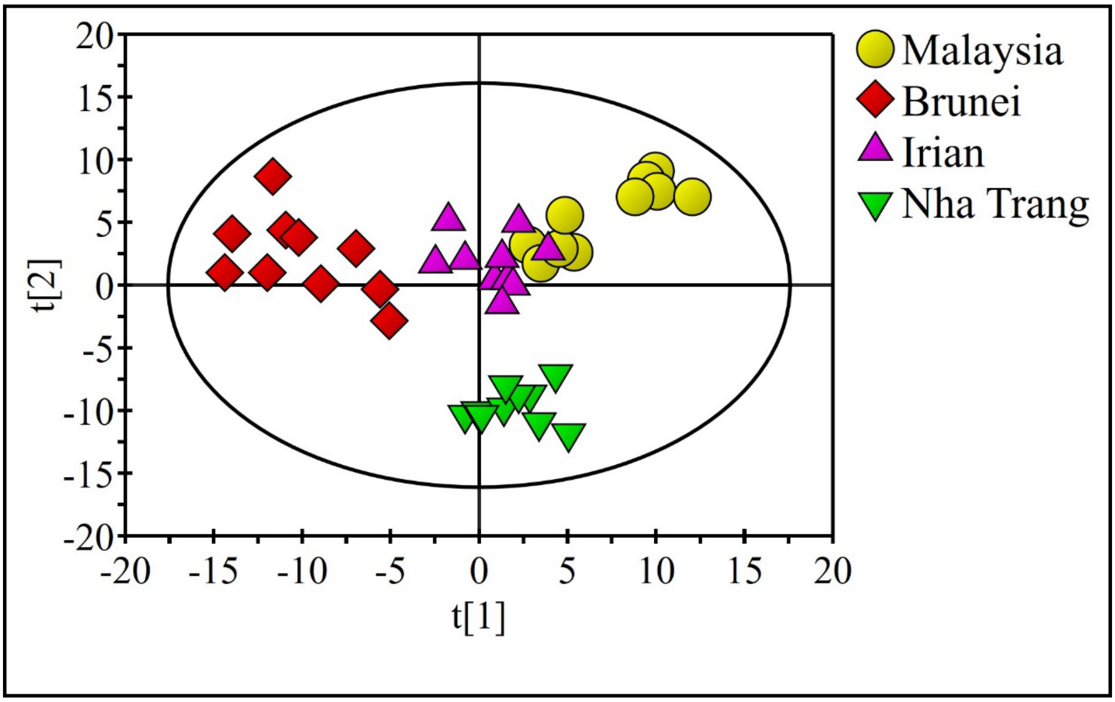

(a)

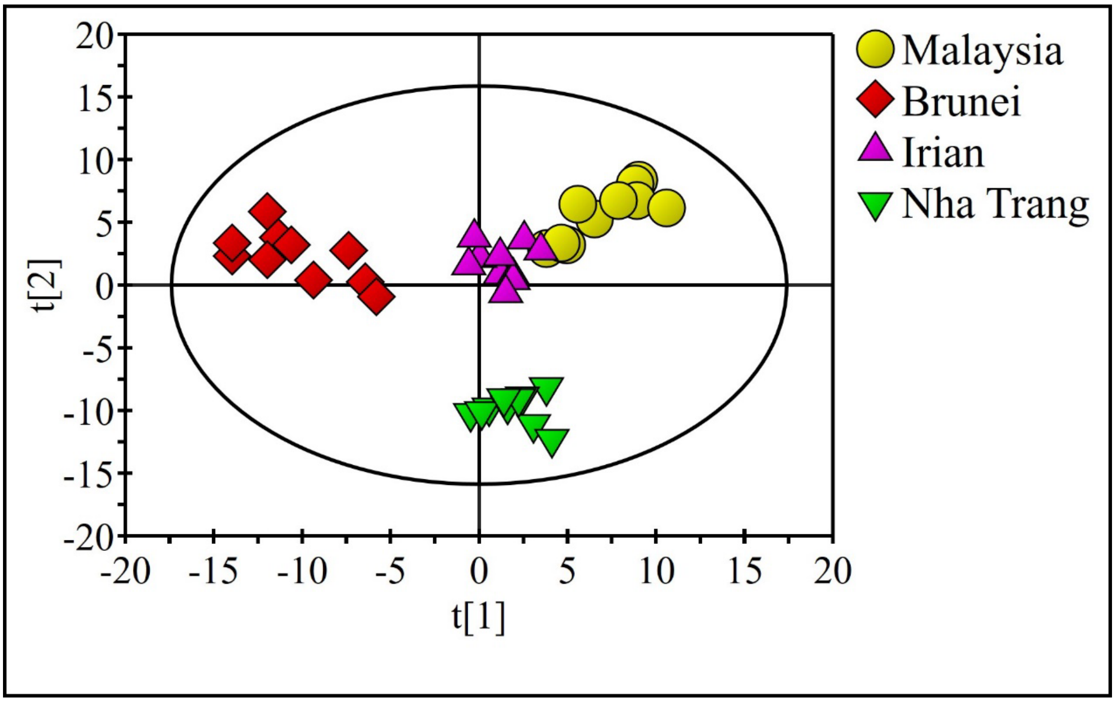

(b)

Figure S2. PCA (a) and PLS-DA (b) score plots of agarwood samples from different geographical origins based on their detected metabolites using SPME-GC/TOFMS (n = 10). Yellow circles, Brunei group; green inverted triangles, Nha Trang group; purple triangles, Malaysia group; red diamonds, Irian group.

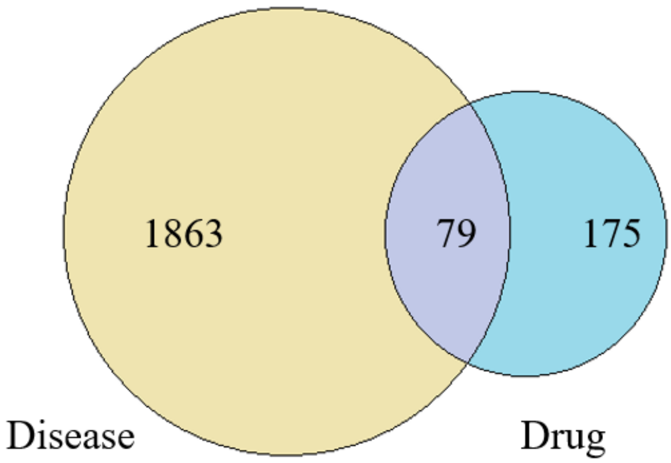

Figure S3. Overlapping genes (79) between those (1,942) related to anxiety disorders and those (254) related to the differential sesquiterpenes in agarwood.

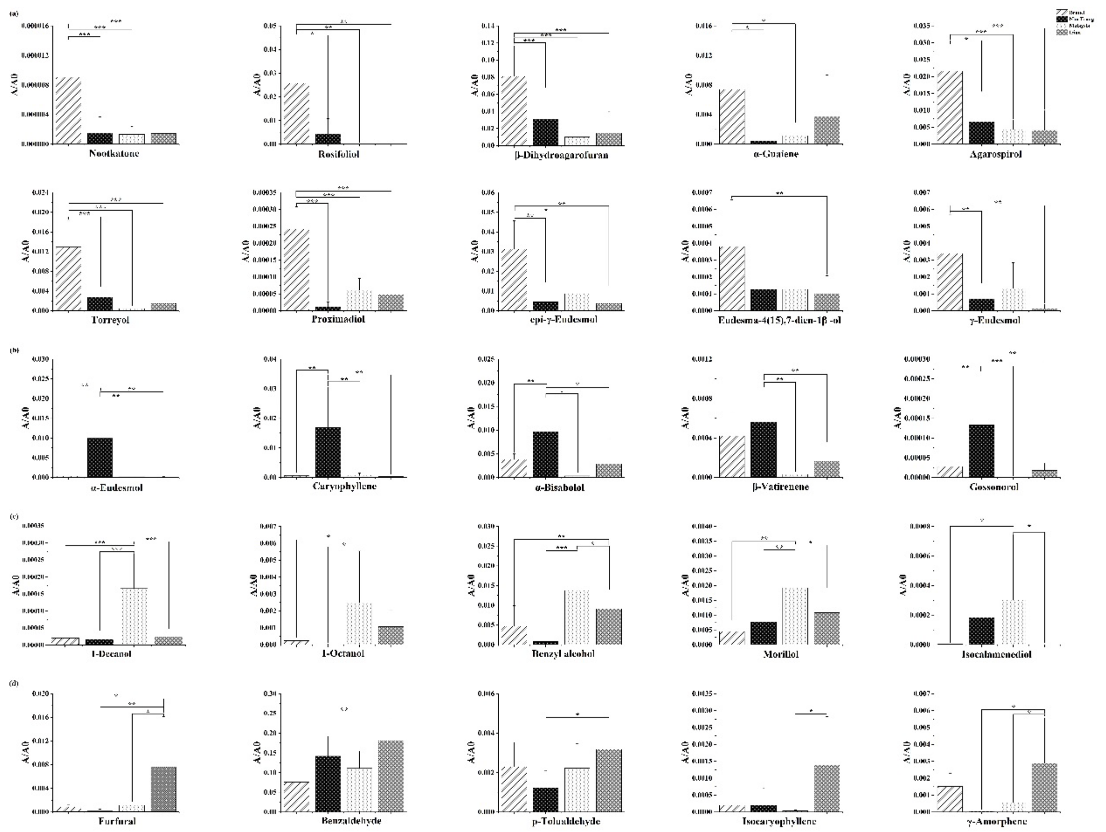

Figure S4. Histogram of the differential compounds among different agarwood groups. Data are presented as the mean ± SD, n = 10 for each group. (a) Brunei group; (b) Nha Trang group; (c) Malaysia group; (d) Irian group. Data are presented as the mean ± SD (n=10). p values were determined using one-way ANOVA analysis. * $p < 0.05$, *** $p < 0.001$.

Table S1. List of the detected volatile compounds in agarwood samples.

No.	Name	R.T. (s)	Retention Index [1]	Lib. RI [2]	Formula	Similarity [2]	Typical fragment ions (*m/z*)
**Furan**							
1	2-Amylfuran	668.09	1235	1231 ± 9(167)	$C_9H_{14}O$	789	53.02、38.98、81.03、95.7、138.12
2	(2R,5S)-2-Methyl-5-(prop-1-en-2-yl)-2-vinyltetrahydrofuran	692.23	1248	1220 ± 17(5)	$C_{10}H_{16}O$	703	54.99、67.04、82.07、95.07、110.08、137.10
3	Furfural [3]	1090.98	1479	1462 ± 11(285)	$C_5H_4O_2$	857	39.97、95.01、96.01
4	2-Acetylfuran	1136.54	1508	1499 ± 10(129)	$C_6H_6O_2$	752	39.00、95.08、96.03、110.12
5	2-Acetyl-5-methylfuran	1321.99	1637	1606 ± 13(26)	$C_7H_8O_2$	714	53.02、81.07、109.02、124.04
6	2-Butanoyl-5-methylfuran	1471.88	1749	1748 ± 0(1)	$C_9H_{12}O_2$	815	52.97、109.06、124.08、137.11、152.13
7	2(5H)-Furanone	1499.79	1770	1742 ± 21(20)	$C_4H_4O_2$	811	28.95、36.99、54.98、84.01
8	Denderalasin	1715.16	1942	1954 ± 0(3)	$C_{15}H_{22}O$	852	69.03、81.03、136.13、175.11、203.15、218.17
**Alcohols**							
9	Methanol [3]	169.57	911.1	903 ± 8(35)	CH_4O	873	28.95、30.97、31.97
10	Ethanol [3]	199.56	943.8	932 ± 8(174)	C_2H_6O	957	30.97、42.97、45.00、46.00
11	Isopropyl Alcohol [3]	192.19	936.6	927 ± 15(71)	C_3H_8O	933	28.97、42.99、45.00、59.02
12	tert-Pentanol	288.12	1019	1008 ± 12(27)	$C_5H_{12}O$	772	43.01、55.04、59.03、73.05
13	3-Buten-2-ol, 2-methyl-	337.08	1057	1038 ± 11(48)	$C_5H_{10}O$	804	42.99、53.01、59.02、71.04
14	2-Butanol, 2,3-dimethyl-	461.58	1118	1100 ± 1(3)	$C_6H_{14}O$	844	31.02、41.02、59.01、87.07
15	2-Pentanol, 2-methyl-	473.50	1125	1101 ± 9(11)	$C_6H_{14}O$	864	30.98、41.01、45.00、59.03、69.04、87.07
16	2-Pentanol	495.43	1146	1119 ± 10(90)	$C_5H_{12}O$	793	45.00、55.03、73.04
17	1-Butanol	538.96	1168	1142 ± 11(292)	$C_4H_{10}O$	925	31.02、41.00、43.01、56.03、73.05、74.05
18	2-Pentanol, 3-methyl-	579.07	1189	1181 ± 21(6)	$C_6H_{14}O$	812	45.00、56.03、69.04、87.09、101.05
19	1-Pentanol	736.54	1271	1250 ± 9(278)	$C_5H_{12}O$	793	30.97、42.01、55.02、57.03、70.06
20	Cyclopentanol	821.99	1318	1312 ± 8(44)	$C_5H_{10}O$	801	43.99、57.00、58.01、67.03、86.06
21	2-Propanol, 1-butoxy-	890.95	1357.6	1353 ± 10(2)	$C_7H_{16}O_2$	784	30.96、45.00、57.04、87.07、102.05
22	1-Hexanol	909.21	1367	1355 ± 7(347)	$C_6H_{14}O$	833	43.01、56.03、69.05、84.08
23	Morillol [3]	1064.38	1462	1450 ± 7(311)	$C_8H_{16}O$	889	29.00、43.03、57.03、72.04、85.09
24	1-Heptanol	1073.96	1468	1453 ± 8(127)	$C_7H_{16}O$	818	43.01、56.03、69.05、70.06、83.03、98.10
25	1-Hexanol, 2-ethyl-	1125.89	1501	1491 ± 5(154)	$C_8H_{18}O$	912	57.04、70.06、83.07、98.10、112.12、130.08
26	1-Octanol [3]	1227.87	1572	1557 ± 8(338)	$C_8H_{18}O$	828	56.03、69.05、70.06、84.08、112.13、130.08
27	1,2-Butanediol	1280.54	1608	1563 ± 0(1)	$C_4H_{10}O_2$	791	30.97、59.02、61.00
28	1-Nonanol	1371.37	1674	1660 ± 7(95)	$C_9H_{20}O$	814	30.99、43.03、56.04、70.06、83.08、98.11
29	1-Decanol [3]	1508.10	1777	1760 ± 9(70)	$C_{10}H_{22}O$	726	40.98、55.04、70.06、83.10、97.09、112.08

30	2-Phenylisopropanol	1510.84	1779	1773 ± 5(6)	$C_9H_{12}O$	868	43.01、77.03、91.05、118.08、121.07、136.09
31	Benzenemethanol, α-methyl-	1580.20	1833	1801 ± 19(37)	$C_8H_{10}O$	863	50.99、77.03、79.04、107.05、122.07、136.09
32	p-Cymenol-8	1631.81	1874	1852 ± 12(179)	$C_{10}H_{14}O$	789	43.01、91.02、115.06、135.17、150.10
33	Benzyl alcohol[3]	1661.48	1897	1870 ± 14(323)	C_7H_8O	870	50.99、77.02、79.04、91.05、107.05、108.06
34	Phenylethyl alcohol[3]	1704.02	1933	1906 ± 15(423)	$C_8H_{10}O$	931	38.98、65.02、91.05、92.06、122.07
35	1-Dodecanol	1768.89	1988	1969 ± 9(85)	$C_{12}H_{26}O$	729	43.05、55.03、83.07、97.07、140.16、168.21
36	Anise alcohol	2131.23	2318	2296 ± 8(4)	$C_8H_{10}O_2$	795	38.99、77.02、94.04、109.06、121.06、138.06
37	2-(4-Methoxyphenyl)ethanol	2182.82	2369	2341 ± 14(3)	$C_9H_{12}O_2$	875	30.00、78.04、121.06、122.07、152.08
**Aldehydes**							
38	Acetaldehyde	105.49	642.3	702 ± 12(82)	C_2H_4O	841	28.95、40.99、43.01、43.99
39	2-Propenal[3]	142.77	847	850 ± 10(17)	C_3H_4O	809	28.95、35.93、36.97、54.99、56.00
40	Butanal, 2-methyl-	175.28	917.5	914 ± 8(126)	$C_5H_{10}O$	747	28.95、41.00、57.04、58.02、86.06
41	Butanal, 3-methyl-	178.60	921.3	918 ± 7(202)	$C_5H_{10}O$	831	41.00、43.00、57.04、58.02、71.03、86.06
42	Hexanal[3]	393.73	1093	1083 ± 8(553)	$C_6H_{12}O$	782	41.00、43.99、56.03、57.03、58.02、72.04
43	Heptanal[3]	588.11	1194	1184 ± 9(257)	$C_7H_{14}O$	735	41.00、55.02、70.06、81.05、96.09、114.10
44	2-Butenal, 3-methyl-	629.89	1203	1214 ± 12(16)	C_5H_8O	793	28.98、41.00、55.02、84.04
45	Octanal[3]	782.62	1295	1289 ± 9(377)	$C_8H_{16}O$	756	43.02、55.01、70.02、84.08、100.08、110.11
46	Nonanal[3]	962.49	1398	1391 ± 8(461)	$C_9H_{18}O$	783	41.00、57.04、70.06、98.10、114.11、124.13
47	cis-2-Nonenal	1188.88	1545	1502 ± 10(61)	$C_9H_{16}O$	759	41.00、55.02、70.03、83.05、96.09、111.11
48	Benzaldehyde[3]	1172.22	1533	1520 ± 14(471)	C_7H_6O	905	49.98、50.99、77.02、105.03、106.04
49	p-Tolualdehyde[3]	1343.85	1654	1653 ± 5(16)	C_8H_8O	888	38.99、63.01、65.02、91.05、119.05、120.06
50	trans-2-Decenal	1352.21	1659	1644 ± 11(84)	$C_{10}H_{18}O$	776	28.99、43.00、55.02、70.03、83.04、110.10
51	Benzaldehyde, 2-hydroxy-	1394.88	1691	1672 ± 13(30)	$C_7H_6O_2$	890	38.98、65.02、93.02、104.02、121.03、122.04
52	3,4-Dimethylbenzaldehyde	1545.99	1804	1790 ± 0(1)	$C_9H_{10}O_3$	781	77.03、91.03、105.06、133.05、134.07
53	m-Anisaldehyde	1691.60	1922		$C_8H_8O_2$	811	65.02、77.03、92.02、107.05、135.05、136.06
54	Z-12-Tetradecenal	1755.89	1977	1994 ± 0(1)	$C_{14}H_{26}O$	734	55.03、69.06、98.07、111.10、192.15、210.15
55	Benzaldehyde, 4-methoxy-	1840.00	2050	2011 ± 21(45)	$C_8H_8O_2$	873	63.00、77.03、92.02、107.05、135.05、135.06
56	trans-Cinnamaldehyde	1857.12	2065	2040 ± 8(7)	C_9H_8O	742	50.99、77.01、78.03、103.05、131.05、132.07
57	Vanillin	2411.74	2605	2568 ± 13(129)	$C_8H_8O_3$	842	53.00、81.03、109.03、123.05、151.05、152.05
**Ketones**							
58	Acetone[3]	131.59	810.5	819 ± 6(113)	C_3H_6O	946	41,97、42.98、58.01
59	2-Butanone	166.58	896.1	907 ± 11(109)	C_4H_8O	853	42.99、57.01、72.04
60	2-Butanone, 3-methyl-	186.56	920.9	936 ± 9(21)	$C_5H_{10}O$	902	41.01、42.99、70.99、86.06
61	2-Pentanone	233.65	974.1	981 ± 11(141)	$C_5H_{10}O$	793	41.02、43.00、58.02、70.99、86.07
62	5-Hexen-2-one	486.38	1131	1137 ± 2(2)	$C_6H_{10}O$	868	38.99、41.01、42.99、55.03、83.03、98.08
63	3-Heptanone	519.89	1149	1161 ± 9(24)	$C_7H_{14}O$	811	41.01、57.03、72.03、85.06、114.10

64	3-Hexanone, 2,5-dimethyl-	551.56	1175	1145 ± 0(3)	$C_8H_{16}O$	830	41.00、43.01、57.04、71.03、85.05、128.12
65	3-Heptanone, 2-methyl-	562.96	1171	1179 ± 9(7)	$C_8H_{16}O$	903	29.00、43.02、57.05、71.03、85.06、128.12
66	2-Heptanone	579.42	1180	1182 ± 8(180)	$C_7H_{14}O$	837	29.00、43.00、58.02、71.05、114.11
67	Cyclopentanone, 2-methyl-	593.37	1187	1177 ± 3(8)	$C_6H_{10}O$	827	27.98、42.02、55.01、69.02、98.06
68	Cyclopentanone, 3-methyl-	635.61	1210	1225 ± 1(3)	$C_6H_{10}O$	912	27.98、42.02、55.01、69.02、98.07
69	4-Octanone	666.59	1235	1234 ± 6(6)	$C_8H_{16}O$	768	41.00、43.01、57.04、71.02、85.07、128.12
70	2-Heptanone, 4,6-dimethyl-	693.56	1242	1262 ± 0(1)	$C_9H_{18}O$	817	42.98、58.02、69.05、84.08、85.07、142.04
71	2-Octanone	778.61	1293	1287 ± 8(70)	$C_8H_{16}O$	743	41.00、42.99、58.01、71.05、85.08、128.12
72	2-Propanone, 1-hydroxy-	830.33	1321	1303 ± 12(61)	$C_3H_6O_2$	831	30.97、42.96、74.02
73	Sulcatone	874.09	1347	1338 ± 9(226)	$C_8H_{14}O$	879	42.98、55.02、58.01、69.05、108.09、126.10
74	2-Pentanone, 4-hydroxy-4-methyl-	935.25	1385	1358 ± 14(34)	$C_6H_{12}O_2$	821	42.98、58.01、59.02、98.06、101.05
75	2-Nonanone	957.40	1395	1390 ± 7(127)	$C_9H_{18}O$	748	58.01、71.05、83.06、85.07、127.13、142.04
76	2,5-Hexanedione	1155.98	1522	1500 ± 4(6)	$C_6H_{10}O_2$	827	42.98、57.03、71.02、99.04、114.06
77	6-Methyl-3,5-heptadiene-2-one	1281.86	1609	1602 ± 0(18)	$C_8H_{12}O$	821	42.99、53.01、79.01、81.06、109.06、124.09
78	Acetophenone	1358.18	1664	1647 ± 13(126)	C_8H_8O	888	38.98、42.98、50.99、77.03、105.03、120.06
79	1-Propanone, 1-phenyl-	1464.40	1742	1715 ± 19(7)	$C_9H_{10}O$	767	50.99、77.02、105.03、134.08
80	α-Phenylacetone	1465.31	1744	1710 ± 1(6)	$C_9H_{10}O$	890	42.98、65.01、91.05、92.06、134.08
81	Ethanone, 1-(3-methylphenyl)-	1525.89	1790	1795 ± 9(2)	$C_9H_{10}O$	832	50.99、65.02、91.05、119.05、134.07
82	p-Hydroxyacetophenone	1558.69	1816	1788 ± 2(4)	$C_8H_8O_2$	795	38.98、52.98、65.02、93.02、121.03、136.05
83	2-Butanone, 4-phenyl-	1634.25	1875	1867 ± 16(6)	$C_{10}H_{12}O$	822	42.98、77.03、91.05、105.07、133.04、148.09
84	2,2-Dimethyl-3-vinylcyclopentanone	1687.30	1918		$C_9H_{14}O$	885	55.03、67.04、81.05、96.06、123.08、138.11
85	γ-Resacetophenone	1932.54	2132		$C_8H_8O_3$	833	52.92、81.05、137.02、152.05
86	Benzalacetone	1945.13	2144	2099 ± 5(4)	$C_{10}H_{10}O$	721	50.99、63.00、77.01、103.06、131.06、146.08
87	4-Acetylanisole	1971.54	2168	2132 ± 17(8)	$C_9H_{10}O_2$	837	49.99、63.00、64.01、92.00、135.05、150.07
88	2,3,6-Trimethylacetophenone	2030.72	2222		$C_{10}H_{10}O_2$	762	65.02、77.02、91.02、119.03、147.08、162.09
89	2-Butanone, 4-(4-methoxyphenyl)-	2132.54	2319	2236 ± 0(1)	$C_{11}H_{14}O_2$	764	65.02、77.03、91.05、121.07、135.05、178.10
90	Benzophenone	2315.77	2503	2450 ± 16(6)	$C_{13}H_{10}O$	855	50.99、77.03、105.04、152.09、182.08
**Acids**							
91	Acetic acid [3]	1083.69	1474	1449 ± 13(380)	$C_2H_4O_2$	869	42.98、44.96、59.99
92	Formic acid [3]	1173.27	1534	1503 ± 18(16)	CH_2O_2	843	28.96、44.97、45.98
93	Propanoic acid [3]	1211.64	1560	1535 ± 11(130)	$C_3H_6O_2$	901	28.98、44.96、55.98、56.99、73.01、74.02
94	Isobutyric acid [3]	1243.42	1582	1570 ± 12(142)	$C_4H_8O_2$	812	43.03、55.04、73.01、88.04
95	Butanoic acid [3]	1337.98	1650	1625 ± 12(290)	$C_4H_8O_2$	815	41.00、41.98、43.01、59.99、73.01、88.04
96	Isovaleric acid [3]	1393.70	1690	1666 ± 11(258)	$C_5H_{10}O_2$	793	41.00、43.01、44.98、59.99、87.03、101.06
97	Pentanoic acid [3]	1485.73	1760	1733 ± 13(119)	$C_5H_{10}O_2$	818	44.97、55.00、60.00、73.01、87.03、101.06
98	2-Methylvaleric acid [3]	1521.59	1787	1764 ± 9(7)	$C_6H_{12}O_2$	705	44.97、55.00、74.02、87.04、101.06、117.08

99	2-Butenoic acid, 2-methyl-, (Z)-	1554.72	1813	1816 ± 34(4)	$C_7H_{12}O_3$	765	29.01、43.01、55.03、85.05、100.05、129.09
100	Isohexanoic acid	1570.12	1825	1803 ± 12(11)	$C_6H_{12}O_2$	801	43.01、57.04、60.00、73.01、74.02、101.08
101	Hexanoic acid [3]	1623.06	1866	1846 ± 12(326)	$C_6H_{12}O_2$	819	44.97、60.00、73.01、87.04、99.07
102	Hexanoic acid, 2-ethyl-	1747.33	1970	1960 ± 9(27)	$C_8H_{16}O_2$	879	57.04、73.01、88.04、101.06、116.08、144.12
103	α-Ethylcaproic acid	1747.33	1970	1954 ± 11(27)	$C_8H_{16}O_2$	712	41.00、57.04、73.01、88.04、101.06、116.08
104	Heptanoic acid [3]	1753.29	1975	1950 ± 15(90)	$C_7H_{14}O_2$	844	60.00、70.06、73.01、87.03、101.06、130.10
105	Octanoic acid [3]	1876.84	2082	2060 ± 15(265)	$C_8H_{16}O_2$	753	43.03、55.03、60.00、73.02、101.06、115.07
106	Decanoic acid [3]	2109.92	2297	2276 ± 14(177)	$C_{10}H_{20}O_2$	735	60.00、73.01、87.03、129.09、143.10、172.15
107	Benzoic acid [3]	2277.63	2464	2412 ± 13(59)	$C_7H_6O_2$	899	49.99、50.99、77.02、104.99、122.04
108	Hydrocinnamic acid	2467.83	2670	2637 ± 14(8)	$C_9H_{10}O_2$	704	50.99、77.03、91.04、104.05、150.06
**Esters**							
109	Propanoic acid, ethenyl ester	258.90	1005	960 ± 0(1)	$C_5H_8O_2$	769	28.98、44.02、57.02、100.08
110	Acetic acid, butyl ester	379.76	1084	1074 ± 8(131)	$C_6H_{12}O_2$	756	41.00、42.98、56.03、61.00、73.01、115.07
111	Caproic acid methyl ester	592.85	1196	1184 ± 7(74)	$C_7H_{14}O_2$	746	54.94、59.01、74.02、87.04、99.09、101.06
112	1-Methoxy-2-propyl acetate	679.69	1241	1233 ± 5(2)	$C_6H_{12}O_3$	774	28.96、72.04、87.03、117.08
113	Heptanoic acid, methyl ester	780.41	1294	1284 ± 8(21)	$C_8H_{16}O_2$	782	28.96、42.98、74.02、87.03、101.5、113.05、14
114	Ethanedioic acid, dimethyl ester	934.40	1380	1395 ± 13(7)	$C_4H_6O_4$	779	45.01、59.03、118.7
115	Propanedioic acid, dimethyl ester	1165.53	1529	1489 ± 10(9)	$C_5H_8O_4$	827	58.98、68.96、74.07、101.02、132.12
116	Diethyl malonate	1263.45	1596	1574 ± 5(12)	$C_7H_{12}O_4$	810	42.99、60.00、88.04、115.04、133.05、160.10
117	Allyl nonanoate	1293.71	1618	1610 ± 0(1)	$C_{12}H_{22}O_2$	760	41.01、55.04、71.01、100.05、141.09、183.14
118	γ-Butyrolactone	1310.23	1629	1616 ± 11(20)	$C_4H_6O_2$	722	28.97、42.02、56.04、86.03
119	Butanedioic acid, diethyl ester	1408.38	1700	1681 ± 9(79)	$C_8H_{14}O_4$	916	28.98、54.99、73.01、101.01、129.05
120	Methyl hydrocinnamate	1615.03	1860	1855 ± 2(6)	$C_{10}H_{12}O_2$	805	51.00、65.02、77.03、91.05、104.06、164.09
121	Pantolactone	1859.70	2067	2029 ± 21(13)	$C_6H_{10}O_3$	831	42.98、57.01、71.03、72.04、112.11、130.06
122	1,2-Benzenedicarboxylic acid, ethyl me-	2180.81	2367	2315 ± 0(1)	$C_8H_6O_4$	821	65.02、77.03、92.06、149.03、163.05、208.09
123	Diethyl Phthalate	2214.51	2400	2366 ± 8(22)	$C_{12}H_{14}O_4$	842	65.06、76.06、105.10、149.0、177.12、222.16
124	Dibutyl phthalate	2515.47	2735	2680 ± 13(12)	$C_{16}H_{22}O_4$	859	104.03、121.04、149.03、205.10、223.10、278.1
**Monoterpenoids**							
125	(+)-3-Carene	480.02	1138	1148 ± 0(1)	$C_{10}H_{16}$	731	77.02、79.06、91.05、93.06、121.10、136.13
126	D-Limonene [3]	586.40	1193	1199 ± 6(6)	$C_{10}H_{16}$	771	68.04、79.04、93.06、107.08、121.10、136.13
127	Eucalyptol	612.18	1197	1213 ± 9(356)	$C_{10}H_{18}O$	889	42.96、55.02、81.07、108.09、139.12、154.13
128	trans-9-Methyldecalin	613.81	1207		$C_{11}H_{20}$	753	67.03、81.06、95.08、109.10、137.13、152.16
129	Trimethylenenorbornane	658.29	1230	1243 ± 0(2)	$C_{10}H_{16}$	726	67.04、79.07、95.08、107.09、121.11、136.13
130	Rosoxide	879.93	1348	1350 ± 13(11)	$C_{10}H_{18}O$	756	41.01、55.03、69.01、83.04、139.11、154.14
131	Inden-5(4H)-one, 2,6,7,7a-tetrahydro-4,4-dimethyl-	974.69	1405		$C_{11}H_{16}O$	817	41.01、79.04、93.06、109.09、120.10、164.16

132	Linalool oxide	1049.27	1452	1445 ± 19(174)	$C_{10}H_{18}O_2$	880	68.05、81.06、94.07、109.07、137.10、155.11
133	trans-Linalool oxide (furanoid)	1093.73	1480	1452 ± 11(175)	$C_{10}H_{18}O_2$	736	59.03、68.06、94.07、111.04、137.10、155.10
134	cis-Linaloloxide	1094.90	1481	1465 ± 20(13)	$C_{10}H_{18}O_2$	776	59.03、81.06、94.07、111.08、137.11、155.11
135	Camphor [3]	1150.05	1518	1518 ± 14(209)	$C_{10}H_{16}O$	865	41.00、55.03、81.06、95.08、108.10、152.12
136	Menthyl acetate	1214.02	1562	1560 ± 14(13)	$C_{12}H_{22}O_2$	885	42.98、54.98、81.06、95.08、123.08、138.11
137	trans -α-Dihydroterpineol	1230.25	1573	1560 ± 0(1)	$C_{10}H_{20}O$	721	59.02、81.06、96.09、123.08、141.13
138	Fenchol	1259.45	1593	1576 ± 0(1)	$C_{10}H_{18}O$	826	42.99、71.04、81.06、93.07、111.08、154.13
139	L-4-terpineneol	1286.19	1612	1593 ± 0(1)	$C_{10}H_{18}O$	740	43.02、71.04、93.08、111.08、136.12、154.13
140	Ipsdienol	1320.24	1637	1631 ± 47(2)	$C_{10}H_{18}O$	805	41.00、、67.04、85.05、109.07、134.11
141	Spiro[2.5]octane, 5,5-dimethyl-4-(3-oxo-butyl)-	1329.44	1643		$C_{14}H_{24}O$	808	45.00、107.07、150.14、175.15、193.15、208.18
142	γ-Terpineol, dihydro-	1342.32	1653	1636 ± 15(2)	$C_{10}H_{20}O$	735	41.00、55.03、71.03、95.08、123.12、138.14
143	dl-Menthol	1347.61	1656	1637 ± 6(53)	$C_{10}H_{20}O$	931	41.00、71.03、95.08、123.11、138.14、154.13
144	α-Citral	1470.21	1748	1732 ± 6(16)	$C_{10}H_{16}O$	765	41.00、69.05、84.05、94.05、137.13、152.12
145	4-Camphenylbutan-2-one	1512.60	1780		$C_{14}H_{22}O$	771	105.06、133.11、148.13、173.14、191.15、206.1
146	Anethole	1587.33	1839	1817 ± 5(9)	$C_{10}H_{12}O$	791	51.02、77.03、91.04、105.06、133.11、148.11
147	Ionone	1602.85	1851	1844 ± 2(2)	$C_{13}H_{20}O$	720	42.99、93.06、121.12、136.13、177.13、192.12
148	Geranyl acetone	1624.55	1868	1859 ± 9(137)	$C_{13}H_{22}O$	899	42.98、69.05、107.09、136.13、151.12、194.16
149	1,3,3-Trimethyl-2-(2-methyl-cyclopro-pyl)-cyclohexene	1786.53	2003		$C_{13}H_{22}$	718	93.07、107.08、121.10、135.13、163.12、178.14
150	(8R,8aS)-8,8a-Dimethyl-3,4,6,7,8,8a-hexa-hydronaphthalen-2(1H)-one	1832.85	2044	2052 ± 0(1)	$C_{12}H_{18}O$	814	79.04、93.07、107.09、136.11、163.12、178.14
151	1,5-Cycloundecadiene, 9-(1-methylethyli-dene)-	1897.54	2100		$C_{14}H_{22}$	772	41.00、93.07、121.09、147.11、175.14、190.16
152	3-Buten-2-one, 4-(2-hydroxy-2,6,6-trime-thylcyclohexyl)-	2048.73	2239		$C_{13}H_{22}O_2$	744	42.98、69.03、109.07、125.10、152.13、210.17
153	(4aS,8R)-4a,8-Dimethyl-4,4a,5,6,7,8-hexa-hydronaphthalen-2(3H)-one	2068.46	2258		$C_{12}H_{18}O$	736	79.03、107.08、121.10、135.13、150.11、178.14
154	Eucarvone	2089.57	2278		$C_{10}H_{14}O$	776	79.06、91.05、107.06、122.09、135.09、150.11
**Agarofurans**							
155	β-Dihydroagarofuran	1427.95	1716	1706 ± 4(5)	$C_{15}H_{26}O$	887	81.06、109.08、137.10、189.18、207.18、222.20
156	4-Epi-cis-Dihydroagarofuran	1509.30	1776		$C_{15}H_{26}O$	873	55.03、69.04、109.08、137.10、189.16、207.17
157	Dihydroagarofuran	1576.58	1829		$C_{15}H_{24}O$	723	95.08、109.09、137.10、149.13、189.17、207.18
158	α-Agarofuran	1654.82	1891	1878 ± 17(3)	$C_{15}H_{24}O$	847	82.03、91.05、123.11、187.16、205.17、220.18
**Agarospiranes**							
159	Agarospirol [3]	1999.42	2193		$C_{15}H_{26}O$	885	59.02、119.08、161.14、189.16、204.19、222.20

160	Hinesol	2012.57	2205	2190 ± 10(4)	$C_{15}H_{26}O$	863	93.05、119.09、147.12、161.14、189.17、204.19
**Guaianes**							
161	Guaia-6,9-diene	1096.59	1482		$C_{15}H_{24}$	744	91.05、105.07、119.09、147.12、161.13、204.19
162	α-Gurjunene [3]	1138.96	1510	1528 ± 7(93)	$C_{15}H_{24}$	890	41.02、55.04、91.05、133.11、189.17、204.19
163	α-Cubebene	1101.97	1486	1463 ± 6(186)	$C_{15}H_{24}$	727	81.04、91.05、105.07、119.09、161.14、204.18
164	β-Cubebene	1236.12	1577	1545 ± 5(133)	$C_{15}H_{24}$	802	81.04、91.05、105.07、119.09、161.13、204.19
165	α-Guaiene	1309.71	1629	1598 ± 6(20)	$C_{15}H_{24}$	850	79.03、93.05、105.07、147.14、189.17、204.19
166	Isoledene	1307.20	1627		$C_{15}H_{24}$	724	91.05、105.07、119.09、133.10、161.13、204.18
167	γ-Gurjunene	1421.13	1710	1674 ± 13(20)	$C_{15}H_{24}$	824	55.03、81.06、107.12、161.14、189.17、204.19
168	Kessane	1491.31	1764		$C_{15}H_{26}O$	924	67.04、81.06、93.06、108.10、126.11、149.13
169	Cyperene epoxide	1622.11	1866		$C_{15}H_{24}O$	745	55.03、91.05、119.09、147.12、205.15、220.18
170	Guaiol	1908.28	2110	2095 ± 9(36)	$C_{15}H_{26}O$	799	59.02、81.05、107.12、161.13、189.16、222.18
171	Cypera-2,4-diene	2017.98	2210		$C_{15}H_{22}$	717	105.05、119.09、145.11、159.13、187.17、202.1
172	Bulnesol	2040.25	2231	2201 ± 15(16)	$C_{15}H_{26}O$	853	59.02、107.09、135.12、161.14、189.17、204.20
173	Curcumenol	2163.72	2350		$C_{15}H_{22}O_2$	704	67.04、105.07、133.10、147.12、189.17、234.15
174	Kessyl acetate	2251.29	2438	2387 ± N/A(1)	$C_{17}H_{28}O_3$	871	108.07、126.11、163.14、205.19、264.21、280.2
**Eudesmanes**							
175	Selina-5,11-diene	1334.09	1647	1620 ± 0(1)	$C_{15}H_{24}$	810	91.05、108.11、121.12、147.12、189.17、204.19
176	α-Selinene [3]	1447.48	1730	1725 ± 12(127)	$C_{15}H_{24}$	893	93.07、133.10、161.14、175.15、189.16、204.19
177	7-epi-α-Selinene	1483.14	1758	1767 ± 7(6)	$C_{15}H_{24}$	785	91.05、107.09、122.11、161.14、189.16、204.18
178	Eudesma-1,4(15),11-triene	1555.63	1813		$C_{15}H_{22}$	860	79.03、91.05、119.09、159.12、187.15、202.17
179	1,7-Dimethyl-4-(propan-2-ylidene)tricyclo[4.4.0.02,7]decan-3-one	1648.59	1887		$C_{15}H_{22}O$	727	68.04、96.05、109.07、121.12、203.16、218.17
180	epi-γ-Eudesmol	1914.69	2116		$C_{15}H_{26}O$	880	91.05、133.11、161.14、189.17、204.20、222.20
181	Rosifoliol [3]	1919.08	2120		$C_{15}H_{26}O$	753	59.02、81.04、108.11、121.12、149.14、164.16
182	Selinenol	1983.58	2179	2177 ± 9(64)	$C_{15}H_{26}O$	779	59.02、105.07、133.11、161.14、189.17、204.19
183	γ-Eudesmol [3]	1990.07	2185	2177 ± 9(64)	$C_{15}H_{26}O$	898	59.05、133.10、161.14、189.17、204.19、222.20
184	α-Eudesmol	2047.76	2239	2229 ± 14(77)	$C_{15}H_{26}O$	811	96.11、149.13、161.14、189.17、204.19、222.20
185	β-Eudesmol	2056.45	2247	2240 ± 18(146)	$C_{15}H_{26}O$	791	108.10、149.14、164.16、189.17、204.19、222.2
186	5β,7βH,10α-Eudesm-11-en-1α-ol	2122.98	2310		$C_{15}H_{26}O$	779	55.01、107.09、161.13、189.17、204.19、222.15
187	Eudesma-4(15),7-dien-1β-ol	2189.88	2376	2370 ± 15(10)	$C_{15}H_{24}O$	757	81.07、91.05、105.07、159.12、177.12、202.17
188	γ-Costol	2258.17	2445		$C_{15}H_{24}O$	813	91.05、105.07、131.10、187.15、205.16、220.18
189	2-(4a,8-Dimethyl-1,2,3,4,4a,5,6,7-octahydro-naphthalen-2-yl)-prop-2-en-1-ol	2369.08	2560	2533 ± 0(4)	$C_{15}H_{24}O$	784	91.05、123.10、145.10、187.16、205.16、220.17
190	Proximadiol	2497.02	2690	2608 ± 0(1)	$C_{15}H_{28}O_2$	797	59.05、81.07、109.07、149.10、189.17、204.15

**Eremophilanes**							
191	Aristolochene	1402.78	1697		$C_{15}H_{24}$	759	91.05、105.07、121.09、161.14、189.17、204.19
192	4a,5-Dimethyl-3-(prop-1-en-2-yl)-1,2,3,4,4a,5,6,7-octahydronaphthalen-1-ol	1521.78	1787		$C_{15}H_{24}O$	887	91.05、131.09、145.11、159.12、187.15、202.17
193	(2R,8R,8aS)-8,8a-Dimethyl-2-(prop-1-en-2-yl)-1,2,3,7,8,8a-hexahydronaphthalene	1560.69	1817	1815 ± 0(1)	$C_{15}H_{22}$	815	91.05、119.09、145.10、161.14、187.15、202.17
194	β-Vetivenene	1594.16	1863	1868 ± 10(6)	$C_{15}H_{22}$	781	91.05、131.09、145.10、160.13、187.15、202.17
195	γ-Vetivenene	1677.28	1910		$C_{15}H_{22}$	840	91.05、131.09、145.11、159.12、187.15、202.17
196	(E)-Isovalencenal	1938.21	2137		$C_{15}H_{22}O$	844	91.05、105.07、119.09、161.13、203.14、218.15
197	4,5-Di-epi-aristolochene	2026.36	2218		$C_{15}H_{24}$	827	93.06、105.07、121.10、161.14、189.17、204.19
198	2(3H)-Naphthalenone, 4,4a,5,6,7,8-hexahydro-4a,5-dimethyl-3-(1-methylethylidene)-, (4ar-cis)-	2294.87	2482	2404 ± 0(1)	$C_{15}H_{22}O$	796	91.05、147.10、161.09、175.14、203.14、218.15
199	Nootkatone [3]	2365.40	2556	2530 ± 15(16)	$C_{15}H_{22}O$	791	91.05、105.07、147.10、169.11、203.14、218.17
**Prezizaane**							
200	Prezizaan-7-ol	2003.61	2197	2188 ± 0(1)	$C_{15}H_{26}O$	793	71.02、109.10、179.10、189.11、204.14、222.15
201	Khusene	1319.77	1637	1609 ± 12(2)	$C_{15}H_{24}$	774	91.05、119.09、119.10、134.11、189.17、204.14
**Bisabolanes**							
202	Himachalene-1,4-diene	1268.61	1600		$C_{15}H_{24}$	825	69.01、91.05、105.07、119.09、161.14、204.19
203	α-Curcumene	1506.35	1775	1777 ± 9(119)	$C_{15}H_{22}$	897	41.00、105.07、119.09、132.10、145.10、202.17
204	cis-Z-α-Bisabolene epoxide	1531.32	1794		$C_{15}H_{24}O$	713	42.98、67.04、93.07、109.07、121.08、220.18
205	α-Dehydro-ar-himachalene	1670.16	1904	1882 ± 0(1)	$C_{15}H_{20}$	771	115.06、128.08、143.08、157.11、185.13、200.1
206	trans-Sesquisabinene hydrate	1791.80	2008	2068 ± 24(2)	$C_{15}H_{26}O$	812	69.05、82.03、119.09、161.12、204.14、222.16
207	Bisabolol oxide II	1953.97	2152	2157 ± 1(5)	$C_{15}H_{26}O_2$	893	42.98、85.06、143.11、161.14、179.15、238.19
208	α-Bisabolol [3]	2033.48	2225	2215 ± 15(76)	$C_{15}H_{26}O$	702	69.04、93.07、109.06、119.10、161.13、204.19
209	Gossonorol	2139.05	2326	2312 ± 0(1)	$C_{15}H_{22}O$	714	42.98、69.06、135.08、157.11、200.14、218.19
210	Spiro[4.5]dec-8-en-7-ol, 4,8-dimethyl-1-(1-methylethyl)-	2124.54	2312		$C_{15}H_{26}O$	776	41.00、84.05、138.11、151.12、179.15、222.20
211	7-epi-cis-sesquisabinene hydrate	2144.64	2331		$C_{15}H_{26}O$	777	41.00、69.05、119.09、161.14、204.19、222.20
**Cadinanes**							
212	γ-Amorphene	1479.00	1754	1719 ± 5(4)	$C_{15}H_{24}$	769	79.04、91.05、105.07、119.09、161.14、204.19
213	β-Cadinene	1492.16	1764	1720 ± 0(1)	$C_{15}H_{24}$	773	91.05、105.08、133.11、161.13、189.17、204.19
214	trans-Calamenene	1579.85	1832	1826 ± 10(23)	$C_{15}H_{22}$	793	115.06、129.08、144.10、159.12、202.17
215	cis-Calamenene	1604.03	1851	1839 ± (144)	$C_{15}H_{22}$	815	115.06、129.08、144.10、159.12、202.17

216	α-Calacorene	1685.05	1917	1919 ± 21(107)	$C_{15}H_{20}$	742	115.07、128.08、142.09、157.11、200.15
217	α-Corocalene	1718.28	1946	2060± 23(3)	$C_{15}H_{20}$	850	115.06、128.07、143.09、157.11、185.13、200.1
218	Torreyol	1958.28	2156	2187 ± 21(102)	$C_{15}H_{26}O$	836	43.02、105.07、121.10、161.13、189.16、204.18
219	Cadalene	2041.24	2232	2233 ± 23(33)	$C_{15}H_{18}$	739	153.08、155.10、168.10、183.13、198.15
220	Isocalamenediol	2219.23	2405		$C_{15}H_{26}O_2$	775	71.05、111.08、155.08、165.13、177.13、223.15
**Aromadendranes**							
221	Alloaromadendrene	1372.07	1675	1665 ± 0(1)	$C_{15}H_{24}$	813	91.05、105.07、133.11、161.14、189.17、204.19
222	(-)-Spathulenol	1496.93	1768		$C_{15}H_{24}O$	774	42.99、91.05、119.09、159.13、205.16、222.16
223	Epiglobulol	1824.45	2036	2025 ± 14(11)	$C_{15}H_{26}O$	725	82.06、109.09、161.11、189.13、204.14、222.16
224	Spathulenol	1968.31	2165	2136 ± 8(220)	$C_{15}H_{24}O$	819	91.05、105.07、119.09、159.13、205.16、220.18
225	1H-Cycloprop[e]azulene, decahydro-1,1,7-trimethyl-4-methylene-	2009.24	2202		$C_{15}H_{24}$	818	41.00、93.06、105.07、161.14、189.17、204.19
226	Aromadendrene oxide-(2)	2093.95	2282	2299 ± 0(1)	$C_{15}H_{24}O$	831	41.02、91.05、133.11、177.12、189.16、220.18
227	Ledene oxide-(II)	2101.08	2289	2269 ± 0(1)	$C_{15}H_{24}O$	811	41.00、91.05、159.09、177.12、202.18、220.18
**Humulanes**							
228	Humulene	1355.26	1662	1667 ± 14(444)	$C_{15}H_{24}$	853	41.01、80.05、93.07、107.9、147.12、204.19
229	Humulene epoxide I	1812.79	2026	2015 ± 8(11)	$C_{15}H_{24}O$	884	80.05、93.06、107.09、121.10、138.11、220.18
230	Humulene epoxide II	1840.76	2051	2071 ± 0(67)	$C_{15}H_{24}O$	849	67.03、96.08、109.09、123.09、138.11、220.18
231	Humulenol-II	2117.00	2304		$C_{15}H_{24}O$	882	41.00、67.04、95.08、109.09、119.09、220.18
**Cedranes**							
232	(-)-α-Cedrene	1181.35	1539	1577 ± 10(47)	$C_{15}H_{24}$	731	93.06、105.08、119.09、161.10、189.16、204.19
233	β-Funebrene	1195.87	1549	1590 ± 4(8)	$C_{15}H_{24}$	754	69.06、93.06、119.09、133.10、161.13、204.19
234	γ-Patchoulene	1348.85	1657	1656 ± 8(2)	$C_{15}H_{24}$	809	41.00、91.06、121.09、161.15、189.16、204.19
235	Diepicedrene-1-oxide	1438.00	1723		$C_{15}H_{24}O$	835	41.00、119.09、150.07、161.15、207.18、220.19
236	Cedrol	1929.36	2129	2116 ± 15(30)	$C_{15}H_{26}O$	845	69.05、95.08、119.11、150.14、207.18、222.20
237	Cedr-8-en-13-ol	2238.80	2425	2359 ± 0(1)	$C_{15}H_{24}O$	817	41.01、91.06、119.11、132.10、189.16、222.20
**Elemanes**							
238	γ-Elemene	1410.75	1703	1642 ± 9(56)	$C_{15}H_{24}$	828	41.00、93.06、121.09、161.14、189.17、222.20
239	β-Elemenone	1869.41	2076	2070 ± 0(1)	$C_{15}H_{22}O$	721	67.05、107.06、121.09、135.09、150.11、218.17
240	Elemol	1894.77	2098	2080 ± 10(94)	$C_{15}H_{26}O$	899	41.00、59.02、93.07、135.12、161.14、189.17
241	β-Elemene	1258.11	1592	1591 ± 9(250)	$C_{15}H_{24}$	877	81.06、107.08、121.10、147.11、189.16、204.19
**Acoranes**							
242	Italicene	1207.37	1557	1536 ± 7(8)	$C_{15}H_{24}$	787	41.02、93.06、119.09、134.1、161.13、204.19
243	Italicene ether	1567.86	1823		$C_{15}H_{24}O$	730	42.98、91.05、105.09、147.12、205.16、220.18
244	Acorenone B	2019.36	2212		$C_{15}H_{24}O$	762	41.00、82.05、109.07、135.09、177.14、220.18
**Longifolanes**							

245	Longifolene	1062.61	1461		$C_{15}H_{24}$	810	79.04、91.05、105.06、161.13、189.15、204.19
246	Isolongifolol	1942.96	2142		$C_{15}H_{26}O$	753	41.00、81.05、95.07、109.09、135.12、222.15
247	Longipinocarveol, trans-	2168.92	2355		$C_{15}H_{24}O$	877	41.00、109.09、159.11、187.15、202.18、220.18
**Germacranes**							
248	Isogermacrene D	1407.40	1700	1665 ± 0(1)	$C_{15}H_{24}$	738	79.04、91.05、105.09、119.11、161.13、204.19
249	Parthenolide	2114.27	2301		$C_{15}H_{20}O_3$	759	81.05、95.06、145.11、190.17、233.15、247.20
250	Ageratriol	2266.06	2453		$C_{15}H_{24}O$	767	55.03、79.07、173.12、201.13、219.14、233.15
**Caryophyllanes**							
251	Isocaryophyllene	1227.20	1571	1587 ± 14(16)	$C_{15}H_{24}$	795	41.02、93.07、133.10、161.14、189.15、204.19
252	β-Caryophyllene [3]	1288.28	1614	1595 ± 16(576)	$C_{15}H_{24}$	796	41.01、79.04、105.07、133.10、161.14、189.16
253	Caryophyllene oxide [3]	1773.45	1992	1989 ± 19(317)	$C_{15}H_{24}O$	787	79.05、93.07、109.09、177.12、205.16、220.18
**Aristolanes**							
254	(-)-Aristolene	1199.69	1552	1572 ± 10(7)	$C_{15}H_{24}$	762	91.05、119.08、133.09、161.14、189.19、204.18
**Copanes**							
255	Copaene	1092.95	1480	1463 ± 6(186)	$C_{15}H_{24}$	718	93.07、105.07、119.09、161.13、189.19、204.19
**Maalianes**							
256	1H-Cyclopropa[a]naphthalene, 1a,2,6,7,7a,7b-hexahydro-1,1,7,7a-tetra-methyl-, [1aR-(1aα,7α,7aα,7bα)]-	1412.84	1704		$C_{15}H_{22}$	874	77.03、91.05、145.10、159.12、187.15、202.17
**Thujopsanes**							
257	Widdrenal	2151.02	2338		$C_{15}H_{22}O$	797	91.05、123.08、133.09、189.16、203.17、218.16
**Lauranes**							
258	Cuparene	1564.85	1820	1826 ± 12(27)	$C_{15}H_{22}$	792	105.05、119.08、132.10、145.10、187.15、202.1
**Other Sesquiterpenes**							
259	α-Ylangene	1121.96	1499	1491 ± 3(69)	$C_{15}H_{24}$	755	93.06、105.05、119.09、161.13、189.17、204.19
260	Isolongifolene	1194.13	1548	1538 ± 14(2)	$C_{15}H_{24}$	782	91.05、133.11、161.13、189.17、175.15、204.18
261	β-Ylangene	1210.33	1559	1589 ± 0(27)	$C_{15}H_{24}$	848	91.05、105.05、120.09、133.11、161.13、204.18
262	α-Bergamotene	1217.36	1564	1584 ± 13(30)	$C_{15}H_{24}$	808	41.00、93.06、107.09、119.09、161.13、204.18
263	Isodaucene	1260.98	1594	1577 ± 9(40)	$C_{15}H_{24}$	785	93.06、121.10、134.11、161.98、189.17、204.18
264	Clovene	1289.95	1615	1617 ± 16(3)	$C_{15}H_{24}$	743	41.01、91.05、105.07、161.12、189.17、204.19
265	Cyclolongifolene oxide, dehydro-	1667.23	1903		$C_{15}H_{22}O$	779	91.05、119.08、133.10、175.14、203.14、218.16
266	Isolongifolene, 4,5,9,10-dehydro-	1726.74	1952		$C_{15}H_{20}$	727	91.05、128.07、143.08、157.11、185.13、200.16
267	Nerolidol	1849.32	2058	2033 ± 14(71)	$C_{15}H_{26}O$	892	69.05、93.07、107.09、161.09、204.19、222.19
268	2,6-Di-tert-butyl-4-hydroxy-4-methylcy-clohexa-2,5-dien-1-one	1921.92	2122	2116 ± 2(4)	$C_{15}H_{24}O_2$	817	57.04、137.08、165.10、180.12、221.16、236.18
269	Muscone	2054.14	2245	2281 ± 0(2)	$C_{16}H_{30}O$	772	41.00、55.03、85.07、125.08、223.18、238.20

270	Valerenal [3]	2065.30	2255	2224 ± 17(2)	$C_{15}H_{22}O$	823	91.06、147.11、175.13、185.07、203.15、218.17
271	Farnesol	2193.47	2379	2350 ± 8(30)	$C_{15}H_{26}O$	865	69.05、81.04、121.10、136.13、161.13、222.19
272	Longipinocarvone	2274.31	2461		$C_{15}H_{22}O$	828	41.00、79.04、134.10、175.14、203.15、218.17
**Others**							
273	2,4-Dimethyl-1-heptene	152.23	880.5	885 ± 0(1)	C_9H_{18}	793	43.02、55.03、57.04、70.06、83.07、126.14
274	Nonane [3]	159.75	900	900	C_9H_{20}	774	42.99、57.04、71.05、85.09、99.11、128.16
275	Benzene [3]	194.53	939.3	957 ± 17(131)	C_6H_6	784	38.98、50.99、77.02、78.03
276	Heptane, 2,2,4,6,6-pentamethyl-	210.99	956.1	949 ± 8(6)	$C_{12}H_{26}$	712	41.00、56.03、57.04、85.09、112.12、170.13
277	Decane [3]	250.58	1001	1000	$C_{10}H_{22}$	807	43.01、57.04、71.07、85.09、113.13、142.16
278	Decane, 4-methyl-	264.47	1005	1030 ± 25(2)	$C_{11}H_{24}$	804	43.04、57.04、71.07、112.12、156.23
279	Acetonitrile [3]	269.71	1008	1013 ± 10(19)	C_2H_3N	726	37.98、38.99、39.99、41.00
280	Decane, 2,4-dimethyl-	310.27	1038		$C_{11}H_{24}$	814	57.04、71.05、85.09、112.12、155.14
281	Toluene [3]	312.69	1041	1042 ± 11(228)	C_7H_8	922	50.99、63.00、65.02、91.05、92.05
282	3,3,5,5-Tetramethylcyclopentene	323.74	1040		C_9H_{16}	733	67.04、81.07、91.05、109.10、124.13
283	Decane, 4-methylene-	345.73	1053		$C_{11}H_{22}$	760	41.01、56.045、69.06、84.09、111.12、154.17
284	Decane, 3-methyl-	357.07	1069	1059 ± 11(2)	$C_{11}H_{24}$	828	57.04、71.06、85.09、126.14、127.15、156.19
285	Heptane, 4-chloro-	366.06	1065	1053 ± 3(4)	$C_7H_{15}Cl$	801	41.02、57.05、70.06、83.08、98.11
286	Undecane [3]	405.20	1100	1100	$C_{11}H_{24}$	857	43.02、57.04、71.07、85.09、113.13、156.19
287	Ethylbenzene [3]	455.40	1126	1129 ± 7(143)	C_8H_{10}	950	50.99、65.02、77.03、78.04、91.05、106.08
288	p-Xylene [3]	471.77	1134	1138 ± 9(150)	C_8H_{10}	888	50.99、63.00、65.02、77.02、91.05、106.08
289	Benzene, 1,3-dimethyl- [3]	484.56	1141	1143 ± 10(154)	C_8H_{10}	889	50.99、53.01、65.02、77.02、91.05、106.07
290	Undecane, 5-methyl-	496.67	1147	1157 ± 0(1)	$C_{12}H_{26}$	803	57.04、71.05、85.09、112.12、170.21
291	Undecane, 4-methyl-	507.23	1152	1147 ± 0(1)	$C_{12}H_{26}$	850	43.01、57.04、71.07、85.09、126.13、170.21
292	Undecane, 2-methyl-	515.61	1157	1155 ± 0(1)	$C_{12}H_{26}$	885	57.04、71.07、85.09、99.11、127.15、170.20
293	o-Xylene [3]	572.41	1186	1186 ± 8(132)	C_8H_{10}	853	50.99、65.02、77.02、91.05、105.07、106.08
294	Dodecane [3]	600.74	1200	1200	$C_{12}H_{26}$	894	43.02、57.04、71.07、85.09、127.15、170.21
295	Pyridine [3]	604.96	1202	1185 ± 10(119)	C_5H_5N	835	38.98、49.98、50.99、52.00、78.02、79.03
296	Benzene, propyl- [3]	616.85	1209	1212 ± 12(68)	C_9H_{12}	844	.00、65.02、78.05、91.05、105.07、120.09
297	Dodecane，2-methyl-	619.06	1210	1200	$C_{13}H_{28}$	814	43.02、57.04、71.07、85.09、141.13、170.21
298	Frontalin	637.14	1211		$C_8H_{14}O_2$	747	42.99、72.05、100.09、112.08、142.10
299	Benzene, 1-ethyl-3-methyl-	644.52	1223	1225 ± 7(46)	C_9H_{12}	770	65.02、77.03、91.06、121.09、136.09
300	Pyridine, 2-methyl-	662.67	1225	1219 ± 9(62)	C_6H_7N	761	51.02、66.03、78.04、92.03、93.05
301	Benzene, (2-methylpropyl)-	676.53	1240	1238 ± 21(18)	$C_{10}H_{14}$	813	51.02、65.02、91.05、92.06、117.08、132.10
302	Mesitylene	685.04	1244	1251 ± 16(62)	C_9H_{12}	858	38.98、77.03、91.05、105.07、120.09
303	1,10-Undecadiene	700.56	1252	1251 ± 1(2)	$C_{11}H_{20}$	721	67.03、69.05、79.04、81.05、96.06、124.10
304	Styrene [3]	718.75	1262	1261 ± 10(102)	C_8H_8	877	51.02、63.03、78.03、103.05、104.06

305	Benzocyclobutene	725.82	1266	1269 ± 3(5)	C_8H_8	758	51.02、77.15、78.17、103.22、104.21
306	2,6-Lutidine	727.68	1267	1254 ± 11(22)	C_7H_9N	757	39.00、66.02、79.04、92.00、107.08
307	Benzene, 1-methyl-3-(1-methylethyl)-	732.97	1269	1269 ± 9(35)	$C_{10}H_{14}$	835	50.99、77.03、91.05、117.08、132.10
308	p-Cymene	741.47	1274	1272 ± 8(543)	$C_{10}H_{14}$	724	65.02、77.03、91.05、117.08、119.09、134.11
309	Cyclohexene, 1-pentyl-	748.76	1278	1287 ± 10(23)	$C_{11}H_{20}$	737	68.04、81.06、96.09、109.09、117.04、152.16
310	Pyrazine, methyl-	753.11	1280	1266 ± 10(129)	$C_5H_6N_2$	723	39.96、53.03、67.00、94.05
311	Benzene, 1,2,4-trimethyl-	755.36	1281	1283 ± 8(67)	C_9H_{12}	922	77.03、91.05、105.07、119.08、133.10、190.11
312	Tridecane [3]	791.35	1300	1300	$C_{13}H_{28}$	806	43.01、57.04、71.07、85.09、113.13、184.22
313	Benzene, 1-methyl-4-propyl-	793.71	1301	1296 ± 16(17)	$C_{10}H_{14}$	837	50.99、77.03、79.04、105.07、134.11
314	Benzene, n-butyl-	810.46	1311	1312 ± 12(47)	$C_{10}H_{14}$	711	65.02、77.02、78.03、91.05、105.07、134.11
315	Benzene, 1-ethyl-3,5-dimethyl-	832.36	1323	1319 ± 6(14)	$C_{10}H_{14}$	832	77.03、91.05、105.07、119.08、134.11
316	Benzene, 1,2,3-trimethyl-	852.93	1335	1340 ± 15(38)	C_9H_{12}	752	50.99、77.03、91.05、105.07、120.09
317	3-Tridecene, (Z)-	859.71	1339	1342 ± 8(34)	$C_{13}H_{26}$	818	41.02、55.03、69.05、85.09、111.12、182.16
318	Benzene, 1-methyl-4-(1-methylpropyl)-	866.95	1343	1315 ± 0(3)	$C_{11}H_{16}$	766	77.03、91.05、105.07、105.05、119.08、148.13
319	Benzene, (1,1-dimethylpropyl)-	872.15	1346	1334 ± 6(4)	$C_{11}H_{16}$	817	77.03、91.05、115.06、119.09、148.12
320	Benzene, 2-ethyl-1,4-dimethyl-	882.15	1352	1340 ± 16(16)	$C_{10}H_{14}$	888	77.03、91.05、121.10、151.08、166.10
321	Anisole	882.73	1353	1345 ± 10(34)	C_7H_8O	754	38.99、65.02、78.03、107.98
322	Formamide, N,N-dimethyl-	885.68	1354	1326 ± 20(18)	C_3H_7NO	733	27.95、29.98、41.99、44.01、73.03
323	Benzene, 1-ethyl-2,4-dimethyl-	886.77	1355	1348 ± 4(12)	$C_{10}H_{14}$	737	65.02、77.04、91.05、105.07、119.09、134.11
324	Benzene, 4-ethyl-1,2-dimethyl-	892.72	1358	1354 ± 10(17)	$C_{10}H_{14}$	702	65.02、77.03、91.05、105.07、119.09、134.11
325	Benzene, 2-ethyl-1,3-dimethyl-	906.16	1366	1359 ± 11(8)	$C_{10}H_{14}$	786	65.02、77.03、91.05、105.07、119.09、134.11
326	Indane	907.21	1366	1365 ± 9(7)	C_9H_{10}	739	63.02、77.03、91.05、115.06、117.07、118.08
327	Tridecane, 3-methyl-	908.10	1367	1366 ± 1(2)	$C_{14}H_{30}$	818	57.07、85.09、99.11、113.13、169.19、198.20
328	Benzene, 1-ethyl-2,3-dimethyl-	910.04	1368	1369 ± 6(12)	$C_{10}H_{14}$	773	65.02、77.03、91.05、105.07、119.09、134.11
329	Benzene, (1,1-dimethylbutyl)-	920.42	1374	1378 ± 0(1)	$C_{12}H_{18}$	781	65.02、77.03、91.05、105.07、119.09、162.15
330	Pyridine, 2-propyl-	947.44	1388	1367 ± 2(3)	$C_8H_{11}N$	827	65.02、79.04、93.06、106.07、120.09
331	Indan, 1-methyl-	952.00	1392	1408 ± 0(1)	$C_{10}H_{12}$	769	63.00、65.02、77.03、91.05、117.07、132.10
332	Tetradecane [3]	966.52	1400	1400	$C_{14}H_{30}$	765	71.07、85.09、99.06、113.13、169.20、198.23
333	Benzene, 1,4-diethyl-2-methyl-	998.08	1420	1425 ± 21(9)	$C_{11}H_{16}$	823	77.03、91.05、105.06、119.09、133.10、148.02
334	Benzene, 1,2,3,5-tetramethyl-	1001.49	1422	1422 ± 23(16)	$C_{10}H_{14}$	872	65.02、77.03、91.05、119.09、134.11
335	2-Propionyl-1-pyrroline	1003.38	1423	1417 ± 17(11)	$C_7H_{11}N$	789	57.06、69.05、70.02、96.05、97.10、125.17
336	Benzene, 1,2,4,5-tetramethyl-	1018.07	1433	1433 ± 16(21)	$C_{10}H_{14}$	854	65.02、77.03、91.05、103.06、119.09、134.11
337	Benzene, 1,3-dichloro-	1035.54	1444	1442 ± 19(16)	$C_6H_4Cl_2$	763	48.97、75.01、111.00、145.97、147.97、150.98
338	Benzene, 1,4-dipropyl-	1071.25	1466	1439 ± 0(3)	$C_{12}H_{18}$	781	77.03、91.05、104.06、119.08、133.10、162.16
339	1H-Indene, 2,3-dihydro-4-methyl-	1080.77	1472	1462 ± 19(7)	$C_{10}H_{12}$	754	50.98、65.00、77.03、91.03、117.07、132.10

340	Benzene, (1-methyl-1-propylpentyl)-	1082.68	1474		$C_{15}H_{24}$	756	91.05、105.07、119.09、147.12、161.13、204.15
341	(E)-1-Phenyl-1-butene	1119.28	1497	1479 ± 0(1)	$C_{10}H_{12}$	791	65.02、77.03、91.05、117.07、132.09
342	Thiazole, 5-ethenyl-4-methyl-	1133.12	1506	1520 ± 8(8)	C_6H_7NS	801	58.02、69.09、97.06、125.07
343	Naphthalene, 1,2,3,4-tetrahydro-1,6,8-trimethyl-	1221.00	1567	1610 ± 0(1)	$C_{13}H_{18}$	845	115.08、128.07、144.10、159.12、174.14
344	1H-Indene, 2,3-dihydro-4,7-dimethyl-	1351.57	1660		$C_{11}H_{14}$	779	91.05、115.06、115.06、128.08、145.10、160.13
345	5H-5-Methyl-6,7-dihydrocyclopentapyrazine	1359.96	1666	1627 ± 19(12)	C8H10N2	829	54.99、78.02、92.07、119.06、133.09、134.10
346	Pentadecane, 2,6,10,14-tetramethyl-	1375.72	1677	1670 ± 2(11)	$C_{19}H_{40}$	781	71.07、85.09、99.08、113.12、183.22、268.00
347	Benzene, 1-ethenyl-4-methoxy-	1398.45	1693	1680 ± 10(5)	$C_9H_{10}O$	890	65.02、77.03、91.05、119.09、134.09
348	Naphthalene [3]	1468.83	1747	1745 ± 18(93)	$C_{10}H_8$	898	64.00、75.00、102.05、128.06
349	Benzo[b]thiophene	1554.50	1812	1751 ± 0(3)	C_8H_6S	734	66.95、88.97、90.04、108.11、134.02
350	Benzene, 1,3,5-trimethyl-2-(1-methylethenyl)-	1568.70	1824		$C_{12}H_{16}$	844	105.06、128.08、130.10、145.11、160.13
351	Veratraldehyde	1573.78	1828	1790 ± 0(1)	$C_9H_{10}O_3$	894	65.02、77.03、95.07、105.06、151.08、166.11
352	Naphthalene, 2-methyl-	1612.94	1858	1858 ± 8(20)	$C_{11}H_{10}$	863	49.99、63.00、115.06、141.08、142.08
353	Dimethyl sulfone	1707.02	1935	1903 ± 9(14)	$C_2H_6O_2S$	773	44.96、47.90、79.00、94.04
354	Naphthalene, 1-ethyl-	1740.69	1964	1943 ± 0(2)	$C_{12}H_{12}$	801	51.00、76.00、115.06、128.07、141.07、156.10
355	Naphthalene, 2,7-dimethyl-	1748.75	1971	1970 ± 0(1)	$C_{12}H_{12}$	764	63.01、77.00、128.08、139.07、141.07、156.10
356	Benzothiazole	1749.55	1971	1958 ± 12(53)	C_7H_5NS	849	63.00、68.96、81.98、91.05、108.00、135.02
357	Creosol	1756.87	1978	1956 ± 12(38)	$C_8H_{10}O_2$	737	55.04、67.01、77.02、95.00、123.05、138.07
358	Butanoic acid, 3-methyl-, 2-phenylethyl ester	1759.82	1980	1963 ± 13(12)	$C_{13}H_{18}O_2$	736	57.05、77.02、85.04、91.05、104.06、149.08
359	Biphenyl	1780.98	1998	1986 ± 12(13)	$C_{12}H_{10}$	798	76.02、102.10、115.11、128.10、154.08、155.08
360	Naphthalene, 1,7-dimethyl-	1784.70	2001	2000 ± 0(3)	$C_{12}H_{12}$	705	62.97、77.02、115.06、128.08、141.11、156.12
361	Naphthalene, 2,3-dimethyl-	1792.68	2008	2008 ± 0(3)	$C_{12}H_{12}$	782	63.00、77.02、115.06、128.07、141.07、156.12
362	Naphthalene, 1,6-dimethyl-	1800.28	2008	2006 ± 0(3)	$C_{12}H_{12}$	791	63.00、77.02、115.06、128.07、141.07、156.10
363	o-Cresol [3]	1815.47	2028	2008 ± 12(48)	C_7H_8O	742	50.99、79.04、90.04、107.04、108.06
364	Phenol [3]	1818.96	2031	2000 ± 15(164)	C_6H_6O	912	49.98、54.99、65.02、66.03、94.04
365	1,4-Dimethylnapthalene	1831.20	2042	2041 ± 0(2)	$C_{12}H_{12}$	708	63.00、77.02、115.06、141.08、156.10
366	Phenol, 2-methoxy-3-methyl-	1876.53	2082	2021 ± 0(1)	$C_8H_{10}O_2$	848	55.02、67.03、77.03、95.02、123.08、138.10
367	Benzenamine, 4-propyl-	1878.43	2084	2050 ± 0(1)	$C_9H_{13}N$	770	77.03、106.07、135.06
368	1,1'-Biphenyl, 3-methyl-	1903.26	2105	2103 ± 1(4)	$C_{13}H_{12}$	712	83.03、91.04、、115.06、128.09、152.09、168.1
369	p-Cresol [3]	1906.60	2109	2080 ± 13(106)	C_7H_8O	755	49.99、77.03、90.04、107.04、107.05、108.06
370	Phenol, p-tert-butyl-	2128.47	2315		$C_{10}H_{14}O$	749	65.02、77.03、91.05、107.06、135.09、150.11
371	2,4-Di-tert-butylphenol	2148.59	2335	2318 ± 10(18)	$C_{14}H_{22}O$	771	41.00、57.04、91.05、115.07、191.15、206.17

372	Coumarin	2298.59	2485.7	2454 ± 6(14)	$C_9H_6O_2$	769	51.03、63.01、89.04、90.05、118.05、146.04

[1] Retention Index displays that each analyte with its absolute retention time correlates to a retention index number. The retention index values can help identify the compound by comparing experimentally found retention indices with known values.

[2] The compound is identified by the commercial libraries from NIST mass spectral library. Only compounds with a similarity > 700 were retained and presented.

[3] The compound is identified by the additional self-built flavour compound database.

Table S2. Sixty-two sesquiterpenes with significant differences among agarwood groups.

NO.	Volatile compound	Brunei agarwood (%)	Nha Trang agarwood (%)	Malay agarwood (%)	Irian agarwood (%)	*p* value
**Agarofurans**						
1	β-Dihydroagarofuran	8.10±3.60^{b}	3.09±3.57^{a}	1.01±1.16^{a}	1.45±2.49^{a}	<0.001
2	4-Epi-cis-Dihydroagarofuran	0.30±0.05^{c}	0.00±0.00^{a}	0.03±0.03^{ab}	0.07±0.11^{b}	<0.001
3	Dihydroagarofuran	0.30±0.05^{b}	0.06±0.16^{a}	0.00±0.00^{a}	0.11±0.16^{a}	<0.001
4	α-Agarofuran	5.06±0.99^{b}	2.14±2.31^{a}	1.15±1.14^{a}	0.62±1.67^{a}	<0.001
**Agarospiranes**						
5	Agarospirol	2.16±0.77^{b}	0.67±0.88^{a}	0.44±0.28^{a}	0.41±0.59^{a}	<0.001
**Guaianes**						
6	Guaia-6,9-diene	0.55±0.40^{b}	0.05±0.08^{a}	0.04±0.03^{a}	0.09±0.15^{a}	<0.001
7	α-Guaiene	0.74±0.77^{b}	0.05±0.11^{a}	0.11±0.16^{a}	0.37±0.56^{ab}	0.02
8	γ-Gurjunene	1.52±0.51^{b}	0.34±0.38^{a}	0.24±0.39^{a}	0.28±0.25^{a}	<0.001
9	Kessane	0.26±0.18^{b}	0.11±0.21^{a}	0.00±0.00^{a}	0.06±0.13^{a}	0.008
10	Cyperene epoxide	0.02±0.03^{b}	0.01±0.01^{ab}	0.00±0.00^{a}	0.00±0.00^{a}	0.021
11	Guaiol	3.89±1.89^{b}	0.02±0.03^{a}	0.04±0.04^{a}	0.14±0.16^{a}	<0.001
12	Cypera-2,4-diene	0.00±0.00^{a}	0.02±0.02^{b}	0.00±0.00^{a}	0.00±0.00^{a}	0.012
**Eudesmanes**						
13	α-Selinene	0.00±0.00^{a}	0.00±0.00^{a}	0.01±0.01^{a}	0.10±0.15^{b}	0.03
14	epi-γ-Eudesmol	3.12±1.45^{b}	0.45±0.94^{a}	0.88±0.92^{a}	0.37±0.86^{a}	<0.001
15	Rosifoliol	2.58±1.88^{b}	0.44±0.63^{a}	0.02±0.02^{a}	0.00±0.00^{a}	<0.001
16	γ-Eudesmol	0.34±0.23^{b}	0.07±0.09^{a}	0.13±0.15^{a}	0.01±0.02^{a}	<0.001
17	α-Eudesmol	0.04±0.04^{a}	0.99±0.93^{b}	0.02±0.02^{a}	0.01±0.01^{a}	<0.001
18	β-Eudesmol	0.28±0.16^{b}	0.02±0.03^{a}	0.11±0.13^{a}	0.01±0.01^{a}	<0.001
19	Eudesma-4(15),7-dien-1β -ol	0.04±0.03^{b}	0.01±0.02^{a}	0.01±0.01^{a}	0.01±0.01^{a}	0.017
20	γ-Costol	0.01±0.01^{b}	0.01±0.01^{ab}	0.00±0.00^{a}	0.00±0.00^{a}	0.015
21	Proximadiol	0.02±0.01^{c}	0.00±0.00^{a}	0.01±0.00^{b}	0.00±0.01^{ab}	<0.001
**Eremophilanes**						
22	β-Vatirenene	0.04±0.02^{b}	0.06±0.03^{b}	0.00±0.00^{a}	0.02±0.02^{a}	<0.001
23	(E)-Isovalencenal	0.03±0.02^{a}	0.00±0.00^{a}	0.00±0.00^{a}	0.13±0.19^{b}	0.023
24	4,5-Di-epi-aristolochene	1.36±0.62^{b}	1.19±0.52^{b}	0.42±0.34^{a}	0.06±0.10^{a}	<0.001
25	Nootkatone	0.00±0.00^{b}	0.00±0.00^{a}	0.00±0.00^{a}	0.00±0.00^{a}	<0.001
**Humulanes**						
26	Humulene epoxide I	0.00±0.00^{a}	0.00±0.00^{a}	0.01±0.01^{b}	0.00±0.00^{a}	0.004
27	Humulene epoxide II	0.04±0.04^{ab}	0.00±0.00^{a}	0.08±0.10^{b}	0.05±0.06^{ab}	0.041
28	Humulenol-II	0.11±0.12^{b}	0.00±0.00^{a}	0.01±0.01^{a}	0.02±0.02^{a}	0.001
**Aromadendranes**						
29	Epiglobulol	0.90±0.61^{a}	2.36±2.16^{b}	0.13±0.11^{a}	0.17±0.40^{a}	0.001
30	Aromadendrene oxide-(2)	0.02±0.01^{b}	0.02±0.01^{b}	0.00±0.00^{a}	0.01±0.01^{b}	<0.001
**Elemanes**						
31	γ-Elemene	1.19±0.81^{b}	0.13±0.33^{a}	0.03±0.04^{a}	0.21±0.49^{a}	<0.001
32	Elemol	0.12±0.04^{b}	0.02±0.06^{a}	0.02±0.02^{a}	0.04±0.04^{a}	<0.001
**Cedranes**						
33	(-)-α-Cedrene	0.14±0.10^{b}	0.02±0.03^{a}	0.06±0.06^{a}	0.06±0.05^{a}	0.005
34	Diepicedrene-1-oxide	1.65±0.77^{b}	1.23±0.74^{b}	0.39±0.45^{a}	0.25±0.47^{a}	<0.001
35	Cedrol	0.01±0.01^{b}	0.00±0.00^{b}	0.09±0.07^{a}	0.03±0.03^{b}	<0.001
36	Cedr-8-en-13-ol	0.05±0.06^{b}	0.01±0.01^{a}	0.00±0.00^{a}	0.01±0.02^{a}	0.002

**Cadinanes**						
37	γ-Amorphene	0.15±0.08^{b}	0.00±0.01^{a}	0.05±0.04^{ab}	0.29±0.27^{c}	0.001
38	β-Cadinene	0.13±0.08^{b}	0.01±0.02^{a}	0.00±0.00^{a}	0.03±0.06^{a}	<0.001
39	trans-Calamenene	0.40±0.22^{c}	0.00±0.00^{a}	0.06±0.05^{a}	0.23±0.22^{b}	<0.001
40	cis-Calamenene	0.26±0.25^{b}	0.00±0.00^{a}	0.02±0.02^{a}	0.03±0.02^{a}	<0.001
41	α-Calacorene	0.13±0.07^{b}	0.07±0.08^{b}	0.01±0.01^{a}	0.09±0.06^{b}	0.002
42	α-Corocalene	0.03±0.01^{a}	0.01±0.01^{a}	0.00±0.00^{a}	0.04±0.05^{b}	0.024
43	Torreyol	1.29±0.53^{b}	0.28±0.19^{a}	0.04±0.04^{a}	0.16±0.13^{a}	<0.001
44	Cadalene	0.07±0.04^{b}	0.00±0.00^{a}	0.02±0.01^{a}	0.05±0.02^{b}	<0.001
45	Isocalamenediol	0.00±0.00^{a}	0.02±0.02^{ab}	0.03±0.04^{b}	0.00±0.00^{a}	0.029
**Bisabolanes**						
46	α-Bisabolol	0.38±0.12^{a}	0.97±0.70^{b}	0.03±0.05^{a}	0.28±0.54^{a}	0.001
47	Gossonorol	0.00±0.00^{a}	0.01±0.01^{b}	0.00±0.00^{a}	0.00±0.00^{a}	<0.001
**Copanes**						
48	Copaene	0.15±0.10^{b}	0.04±0.05^{a}	0.03±0.03^{a}	0.04±0.05^{a}	<0.001
**Caryophyllanes**						
49	β-Caryophyllene	0.06±0.07^{a}	1.68±1.37^{b}	0.07±0.08^{a}	0.03±0.03^{a}	<0.001
50	Isocaryophyllene	0.02±0.03^{a}	0.02±0.05^{a}	0.00±0.00^{a}	0.14±0.17^{b}	0.011
51	Caryophyllene oxide	0.21±0.20^{b}	0.01±0.01^{a}	0.02±0.02^{a}	0.04±0.05^{a}	<0.001
**Thujopsanes**						
52	Widdrenal	0.00±0.00^{a}	0.01±0.01^{b}	0.00±0.00^{a}	0.00±0.00^{ab}	0.035
**Longifolanes**						
53	Longifolene	0.02±0.01^{b}	0.00±0.00^{a}	0.00±0.00^{a}	0.00±0.00^{a}	<0.001
54	Isolongifolol	0.00±0.00^{a}	0.04±0.02^{b}	0.00±0.00^{a}	0.02±0.03^{a}	<0.001
55	Longipinocarveol, trans-	0.02±0.02^{b}	0.00±0.00^{a}	0.00±0.01^{a}	0.01±0.01^{a}	0.004
**Germacrane**						
56	Isogermacrene D	0.05±0.06^{a}	0.05±0.06^{a}	0.27±0.44^{ab}	0.54±0.57^{b}	0.019
57	Parthenolide	0.02±0.01c	0.01±0.02^{b}	0.00±0.00^{a}	0.01±0.01ab	<0.001
**Acoranes**						
58	Italicene ether	0.10±0.05^{a}	0.81±0.49^{b}	0.03±0.03^{a}	0.06±0.10^{a}	<0.001
**Others**						
59	α-Ylangene	0.01±0.03^{a}	0.04±0.06^{a}	0.04±0.03^{a}	0.23±0.35^{b}	0.049
60	Clovene	0.00±0.01^{a}	0.05±0.09^{ab}	0.00±0.00^{a}	0.16±0.21^{b}	0.024
61	Isolongifolene, 4,5,9,10-dehydro-	0.02±0.01^{b}	0.00±0.00^{a}	0.00±0.00^{a}	0.00±0.00^{a}	<0.001
62	Farnesol	0.00±0.00^{a}	0.05±0.04^{b}	0.05±0.07^{b}	0.01±0.01^{a}	0.01

Note: Via Duncan's multiple comparison test, superscripted lowercase letters not shared across columns represent significantly different means across agarwood groups (*P* < 0.05). The average intensity value of each compound is calculated by peak area normalization method. Mean value ± SD are shown (n = 10 for each group).

Table S3. Agarwood samples analyzed in the present study.

Sample Name	Geographic Origin	Sample image
Brunei agarwood	Temburong，Brunei	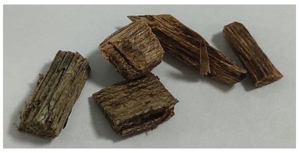
Nha Trang agarwood	Nha Trang, Vietnam	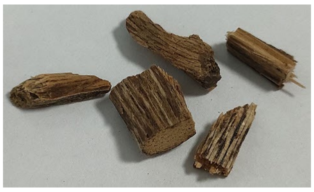
Malaysia agarwood	Kelantan, Malaysia	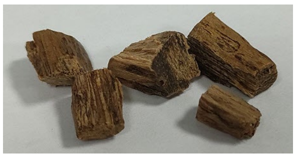
Irian agarwood	Jayapura，Indonesia	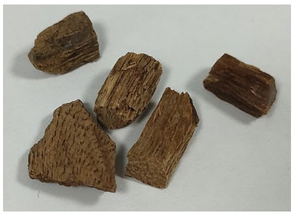
